# Effects of Thermal Processing on the Contents and Bioaccessibility of Polyphenols and Carotenoids in Fresh Waxy Corn

**DOI:** 10.3390/foods15152648

**Published:** 2026-07-28

**Authors:** Hang Liu, Chaoyue Sun, Tianyu Liu, Zijing Gao, Meihong Liu, Jingsheng Liu

**Affiliations:** 1 College of Food Science and Engineering, Jilin Agricultural University, Changchun 130118, China; 2 National Engineering Research Center for Wheat and Corn Deep Processing, Changchun 130118, China

**Keywords:** thermal processing, fresh waxy corn, polyphenol, carotenoid, in vitro digestion

## Abstract

As a typical whole-grain food, fresh waxy corn contains several health-promoting compounds including polyphenols and carotenoids. However, different processing methods can affect the content and health effects of these compounds. This study investigated the effects of atmospheric boiling (AB), high-pressure boiling (HB), high-pressure steaming (HS), and high-speed pulping (HP) on polyphenol and carotenoid content, antioxidant activity, and digestive properties in fresh waxy corn. The results showed that compared with the untreated group, HB could significantly increase the content of polyphenols and carotenoids (*p* < 0.05), with the contents of ferulic acid, quercetin, rutin and proanthocyanidins increasing by 29.06%, 23.71%, 38.15% and 20.82%, while lutein and zeaxanthin increased by 42.31% and 40.43%, respectively. The contents of ferulic acid and zeaxanthin in fresh waxy corn had the greatest correlation with the total antioxidant capacity. In vitro digestion experiments demonstrated that HB treatment improved the bioavailability of polyphenols and carotenoids; however, compared with young adults, polyphenols, including gallic acid, rutin, and quercetin, rather than carotenoids, acted as components with restricted absorption in the elderly gastrointestinal environment.

## 1. Introduction

Whole kernels are abundant in bioactive compounds and have garnered increasing recognition for their potential to mitigate oxidative stress-induced diseases [1]. Fresh waxy corn (*Zea mays* L.S. Kulesh), also known as the ‘golden food’ and ‘longevity food’, is a widely used whole grain [2]. Typical processing techniques for fresh waxy corn, such as steaming/microwave cooking, rapid freezing, hot-air/vacuum drying, milling, and extrusion reforming, significantly modulate the content and physicochemical forms of bioactive compounds [3]. Moderate thermal processing disrupts the ester linkages between cell wall polysaccharides and phenolic acids, facilitating the release of bound phenolics and consequently enhancing both the quantified total phenolic content and the in vitro bioaccessibility [4]. Rapid freezing at −18 °C achieves high phenolic retention (>85%); however, repeated freeze–thaw cycles stimulate polyphenol oxidase activity, leading to partial phenolic browning. Conversely, hot-air drying (>60 °C) causes significant carotenoid degradation due to combined thermal-oxidative effects [5]. Currently, research on fresh waxy corn primarily focuses on flavor changes and starch structural characteristics. However, the transformation patterns of bioactive compounds during thermal processing remain largely unclear, which limits their development and application as ingredients for whole-grain functional foods.

Polyphenols are secondary metabolites that are widely found in plants and are characterized by phenolic functional groups and polyphenolic structures [6]. These compounds exhibit potent antioxidant activities through free radical scavenging, anti-inflammatory effects by suppressing the expression of pro-inflammatory cytokines, and immunomodulatory effects via activation of immune signaling pathways and secretion of immunomodulatory factors. Structurally, carotenoids are tetraterpenoids composed of eight isoprene units linked by a conjugated double-bond system. Since they cannot be synthesized endogenously by the human body, carotenoids must be acquired through dietary intake or supplementation [7]. Conventional assessments of corn quality typically prioritize primary metabolites, such as sugar, starch, and moisture, which primarily govern sensory attributes and basic processing suitability. Consequently, the dynamics of secondary metabolites like carotenoids are frequently overlooked, despite their well-established role in determining the functional and antioxidant value of the grain [8]. Relying solely on traditional parameters may therefore result in an incomplete evaluation of nutritional quality. This limitation becomes particularly evident during thermal processing, which not only alters primary metabolites but also induces structural modifications in bioactive compounds, thereby modulating the extractability of phenolics and promoting their liberation from the food matrix.

In addition, the global population of people aged 65 and over is expected to rise from 761 million in 2021 to 1.6 billion in 2050 (World Health Organization, 2023). This demographic shift poses significant nutritional and public health challenges. Consequently, this study aimed to investigate the physicochemical transformations and digestive profiles of bioactive compounds in fresh waxy corn during thermal processing. Furthermore, age-specific in vitro digestion models were employed to elucidate variations in the digestion and absorption kinetics across different life stages. While existing research on the impact of processing on cereal polyphenol and carotenoid bioavailability is limited and restricted to common varieties, our findings provide a critical theoretical framework for the field. By offering novel insights into the nutritional fate of bioactives, this research facilitates the optimization of processing technologies to enhance nutrient retention and supports the development of personalized functional foods and healthy cereal products.

## 2. Material and Methods

### 2.1. Materials and Reagents

Fresh waxy corn (cv. ‘Jinongnuo 111’ at the milk-ripe stage, with a moisture content of approximately 50%) was harvested in July 2023 from Gongzhuling, Jilin Province, China. It was quick frozen at −80 °C within 4 h after harvesting and stored at −20 °C for subsequent testing. Pancreatic enzyme, pancreatic lipase, trypsin, pepsin, α-amylase, methanol (HPLC grade) were purchased from Sigma Company, methyl tert-butyl ether (HPLC grade) was purchased from Damao Chemical Reagent Factory (Tianjin, China), and acetic acid (HPLC grade) was purchased from Beijing Chemical Plant (Tianjin, China). Foliphenol, pig bile salt, 2,2-biazide (3-hexylbenzothiazolin-6-sulfonic acid), 1,1-diphenyl-2-picrohydrazine, and other chemicals and reagents used in the experiment were of analytical grade and were purchased from Tianjin Fuyu Fine Chemical Co. (Tianjin, China).

### 2.2. Thermal Processes of Fresh Waxy Corn

The corn kernels were obtained after harvesting and divided evenly into five portions (100 g each). To simulate traditional household cooking, the grains were completely cooked during each thermal treatment. During each heat treatment, the corn kernels were thoroughly cooked, and the cooking water was dried together with the kernels in an oven. The specific treatments were as follows:

For the AB treatment group, the kernels were mixed with cold distilled water at a corn-to-water ratio of 1:5 and boiled at 100 °C for 20 min using an induction cooker (C21-SC010). After cooking, the samples were cooled to room temperature.

For the HB treatment group, the kernels were mixed with cold distilled water at a corn-to-water ratio of 1:5 and steamed in a pressure cooker (MY-CS5036P; Midea, Foshan, China) at 115 °C for 20 min. After cooking, the samples were cooled to room temperature.

For the HS treatment group, the kernels were treated in an autoclave (DSX-2803) at 115 °C for 20 min.

For the HP treatment group, the kernels were mixed with distilled water at a ratio of 1:1, homogenized into a slurry using a blender (JYZ-D52), and then steamed at 115 °C for 20 min.

For the RAW group (untreated control), fresh waxy corn kernels were detached, dried in a convection oven at 40 °C, and stored under moisture-proof conditions.

After cooling to room temperature, the samples were dried to a constant weight in a convection oven at 40 °C. The samples were ground using a cryogenic grinder (JXFSTPRP-11; Shanghai Jingxin Technology, Shanghai, China) to pass through an 80-mesh sieve and stored under moisture-proof conditions.

### 2.3. Sample Extraction

#### 2.3.1. Extraction of Free Phenolics and Bound Phenolics

According to the method of Gong K et al. [9], 5 g of corn flour was taken and mixed with 100 mL of 80% methanol. Reaction extraction at room temperature for 2 h, continuous oscillation in a water bath, centrifugation at 6000 r/min for 10 min, and the supernatant was obtained. The procedure was repeated for the residue. The mixed supernatant was evaporated by rotation at 45 °C to obtain free phenolic substances, which were then frozen at −80 °C.

The residue extracted from the above free phenolic substances was hydrolyzed under nitrogen (40–60 s) with 200 mL of 2 M NaOH on a shaking table at room temperature, 200 r/min for 1 h. The mixture was degreased with hexane, the pH was adjusted to 2–3, and then extracted three times with ethyl acetate [10]. The supernatant was combined and evaporated by rotation at 45 °C to obtain the combined phenolic substances, which were frozen in the refrigerator at −80 °C.

#### 2.3.2. Extraction of Carotenoids

According to the method of Zhu Hang et al. [11], lutein and zeaxanthin were prepared as standard solutions with concentrations of 50–200 μg/mL each and analyzed by HPLC, and standard curves were plotted. All sample preparation steps were performed under dim light to minimize carotenoid degradation. Specifically, maize meal and anhydrous ethanol were mixed at a ratio of 1:10 (containing 0.1% antioxidant BHT) and subjected to ultrasonication for 48 min under the following conditions: a temperature of 45 °C, ultrasonic power of 120 W, operating time of 3 s, and interval of 5 s. The mixture was then centrifuged at 5000 r/min for 10 min. The residue was processed again according to the above steps; the ethanol was removed by rotary evaporator, and the carotenoids were obtained and stored in the refrigerator at −80 °C away from light.

### 2.4. Determination of Total Phenolic Compounds

The total phenolic content was determined according to the method described by Gong L [12], and the total phenolic compounds were measured by the Folin–Ciocalteu (FC) method. The gallic acid standard was prepared as a standard solution with concentrations of 10–60 μg/mL, and then 1 mL of FC was diluted with 8 mL of deionized water. One milliliter of the extraction solution and 1 mL of the standard solution were each mixed with 4 mL of deionized water and 5 mL of the Folin-phenol working solution. The absorbance value of the enzyme at 765 nm was measured after the reaction at normal temperature without light for 60 min. Gallic acid was used as the standard, and the total phenolic content was expressed as μg of gallic acid equivalents (GAE) per gram of sample dry weight (DW).

### 2.5. Determination of Total Carotenoid Compounds

The entire extraction process was performed under dim light. A mixture of 30 mL n-hexane-ethanol-acetone (14:6:11, *v*/*v*/*v*) was added to 3 g of corn flour, and the mixture was shaken for 1 h. Then, 2 mL of 40% methanol-potassium hydroxide was added, and the reaction was carried out under nitrogen at room temperature for 16 h. Subsequently, 30 mL of n-hexane was added to distribute the carotenoids; the mixture was shaken for 1 min, 10% sodium sulfate solution was added, and the mixture was diluted to a given volume. The mixture was allowed to stand until the two phases were clearly separated. The upper layer containing the carotenoids was collected, evaporated under nitrogen, dried, and redissolved in 1 mL of chromatographic-grade methanol. The absorbance at 450 nm of the enzyme-labeled instrument was determined. The standard curve was constructed using β-carotene as the reference.

### 2.6. Determination of Monomer Characteristic Phenolic Acids

The same amount of chromatographically pure methanol was filtered through a 0.22 μm pore size organic fiber filter membrane, centrifuged at 12,000 rpm for 10 min, and 20 μL was injected. The extracts were separated using a Zorbax SB-C18 column (150 mm × 4.6 mm, 5 μm), and a high-performance liquid chromatography (HPLC) system (Agilent 1260 Infinity II, Agilent Technologies, Santa Clara, CA, USA) was used for the quantitative analysis of gallic acid, ferulic acid, rutin, proanthocyanidins, and quercetin in corn. The chromatographic column was a Zorbox SBC18 column (150 mm × 4.6 mm, 5 μm) with a diode-array detector set to 280 nm. The mobile phases were 0.5% glacial acetic acid solution (phase B) and methanol (phase A). The elution gradient was as follows: 0–8 min, 5–8% mobile phase A; 8–17 min, 8%–10% mobile phase A; 17–20 min, 10–12% mobile phase A; 20–5 min, 12–15% mobile phase A; 25–33 min, 15–22% mobile phase A; 33–50 min, 22–50% mobile phase A, 50–70 min, 50–80% mobile phase A.

### 2.7. Determination of Monomeric Characteristic Carotenoids

Carotenoids were determined by high-performance liquid chromatography (HPLC) according to the method of Wang Jiaojiao [13] for the quantitative analysis of lutein and zeaxanthin, with the following chromatographic conditions: The chromatographic column was a YMC Carotenoid C30 (4.6 × 250 mm, 5 μm) with a diode array detector (DAD), mobile phase A was methanol, mobile phase B was methyl tert–butyl ether, the ratio of mobile phase A: B = 86:14, the elution time was 20 min, and the sample size was 20 μL. The flow rate was 0.9 mL/min, the column temperature was 30 °C, and the detection wavelength was 450 nm.

### 2.8. Antioxidant Activity Analysis

Free radical scavenging capacity was determined by three methods: DPPH, ABTS^+^, and hydroxyl radical assays.

The blank was prepared without extraction solution (A_d_) or water (A_0_). The radical scavenging activity of the sample was calculated as follows:

The DPPH radical scavenging activity was determined according to the method of Xie [14], with all reagents freshly prepared and protected from light. Briefly, 1 mL of each sample solution at different mass concentrations was thoroughly mixed with 1 mL of 0.2 mmol/L DPPH ethanol solution. The mixture was incubated at room temperature in the dark for 30 min, and the absorbance was measured at 517 nm using a microplate reader and recorded as A_1_. For the blank control, 1 mL of anhydrous ethanol was mixed with 1 mL of 0.2 mmol/L DPPH ethanol solution, incubated under identical conditions, and the absorbance was recorded as A_0_. To correct for sample background interference, 1 mL of each sample solution was mixed with 1 mL of anhydrous ethanol, incubated for 30 min in the dark at room temperature, and the absorbance was recorded as A_b_. The DPPH radical scavenging rate was calculated using Equation (1).

(1) $$\mathrm{DPPH}\;\mathrm{scavenging}\;\mathrm{activity}\;(\%)=(1-\frac{A_{0}-{\;A}_{b}}{A_{0}})\times 100$$

The ABTS^+^ radical scavenging activity was determined as follows. The ABTS^+^ working solution was prepared by mixing 5 mL of 7.4 mmol/L ABTS^+^ solution with 5 mL of 2.6 mmol/L potassium persulfate solution, followed by incubation in the dark at room temperature for 12 h. Prior to the assay, the solution was diluted with 0.1 M phosphate buffer (pH 7.4) to adjust the absorbance at 734 nm to 0.70 ± 0.02, which was recorded as A_0_. For the measurement, 1 mL of each sample solution at different mass concentrations was thoroughly mixed with 4 mL of the ABTS^+^ working solution. After incubation in the dark for 6 min, the absorbance was measured at 734 nm using a microplate reader and recorded as A_1_. The ABTS^+^ radical scavenging rate was calculated according to Equation (2).

(2) $${\mathrm{ABTS}}^{+}\;\mathrm{scavenging}\;\mathrm{activity}\;(\%)=(1-\frac{A_{1}}{A_{0}})\times 100$$

The hydroxyl radical scavenging activity was determined as follows. Solutions of ferrous sulfate, hydrogen peroxide, and salicylic acid were each prepared at a concentration of 8 mmol/L. Briefly, 5.0 mL of each of the ferrous sulfate, hydrogen peroxide, and salicylic acid solutions were added sequentially to 5 mL of each sample solution at varying mass concentrations, and the mixture was thoroughly mixed. After incubation in a water bath at 37 °C for 30 min, the absorbance was measured at 510 nm using a microplate reader and recorded as A_1_. The absorbance of the control mixture, in which the sample solution was replaced with distilled water, was recorded as A_0_. The absorbance of the blank mixture, in which salicylic acid was replaced with distilled water, was recorded as A_2_. The hydroxyl radical scavenging rate was calculated according to Equation (3).

(3) $$\mathrm{Hydroxyl}\;\mathrm{radical}\;\mathrm{scavenging}\;(\%)=(A_{0}-\frac{A_{1}-A_{2}}{A_{0}})\times 100$$

The IC_50_ value was defined as the concentration of sample required to scavenge 50% of the corresponding radicals. IC_50_ values for DPPH, ABTS^+^, and hydroxyl radical scavenging activities were calculated from the concentration–response curves of scavenging activity versus sample concentration.

### 2.9. Test Method for Simulated Gastrointestinal Digestion In Vitro

#### 2.9.1. Simulated Gastrointestinal Digestion In Vitro

The digestion method was adapted from [15] and modified accordingly. The in vitro-simulated digestion was divided into three stages: oral, gastric, and intestinal. The formulations of simulated salivary fluid (SSF), simulated gastric fluid (SGF), and simulated intestinal fluid (SIF) are shown in the Supplementary Materials.

A total of 5 mL of SSF pre-warmed to 37 °C was added to 5 g of corn flour sample, mixed well by vortex stirring, and digested in a 37 °C water bath for 2 min to complete oral digestion. Then, 10 mL of SGF, preheated to 37 °C, was added to the digested samples, and the pH was adjusted (see Tables S1 and S2). The digested samples were transferred to a shaker at 37 °C and 150 rpm to simulate digestion for 2 h, and the samples were removed at 15, 60, and 120 min, respectively, to simulate gastric digestion. The samples were centrifuged at 5000 rpm for 10 min. Three experiments were performed in parallel for each sample. The digestion solution was centrifuged at 5000 rpm for 30 min, and the supernatant (i.e., the released active substance) was collected to minimize enzyme activity and remove the undigested substance. The supernatant was lyophilized to maintain quality and stored in a dry environment. After gastric digestion was completed, 20 mL of SIF preheated to 37 °C was added to the digestion solution, and the pH was adjusted to 7.0. The digestion solution was subjected to simulated digestion on a shaker table at 37 °C and 150 rpm for 4 h, and samples were taken at 60, 120, and 240 min. The samples were centrifuged at 4 °C, 5000 rpm for 10 min. Three parallel experiments were conducted for each sample. After digestion, the samples were centrifuged at 3000× *g* for 10 min, and the supernatant (the active substance released from chyme) was stored at 4 °C. The supernatant was frozen and dried to a constant mass and stored in a dry environment for analysis [16].

#### 2.9.2. Two Kinds of Standardized Static In Vitro Digestive Environment Simulation for Young Adults and Old People

The standardized static in vitro digestion methods were simulated by reference to the two in vitro gastrointestinal digestion environments of young and old people [17,18].

As listed in Table S2, the stimulated digestion conditions were as follows. Oral: Salivary amylase activity increased to 150% in the elderly. Stomach: The frequency and amplitude of peristaltic contraction in the elderly decreased to 80%, the activity of pepsin decreased to 75%, and the pH value increased (2.0–6.2). In the simulated stomach model of the elderly, the stomach contraction was relatively weak, resulting in the fluctuation of the stomach pH value [6], and the secretion of gastric juice (stomach acid) decreased to 85%. Small intestine: The frequency and amplitude of small intestine peristalsis in the elderly were reduced to 80%, trypsin activity was reduced to 50%, and pancreatic lipase activity was reduced to 15% [18]. Pancreatic fluid secretion was reduced to 90%.

### 2.10. Statistical Analysis

All experimental data were expressed as the mean ± standard deviation (SD). Analysis of variance (ANOVA) and principal component analysis (PCA) were performed using SPSS Statistics 23 (IBM Corp., Armonk, NY, USA). For the PCA, principal components were extracted based on the correlation matrix, and all variables were standardized prior to analysis. Data visualization and graphing were conducted using GraphPad Prism 9.5 and OriginPro 2021. A *p*-value < 0.05 was considered statistically significant.

## 3. Results

### 3.1. The Contents of Polyphenols and Carotenoids in Corn by Different Thermal Processing

**Figure 1:**
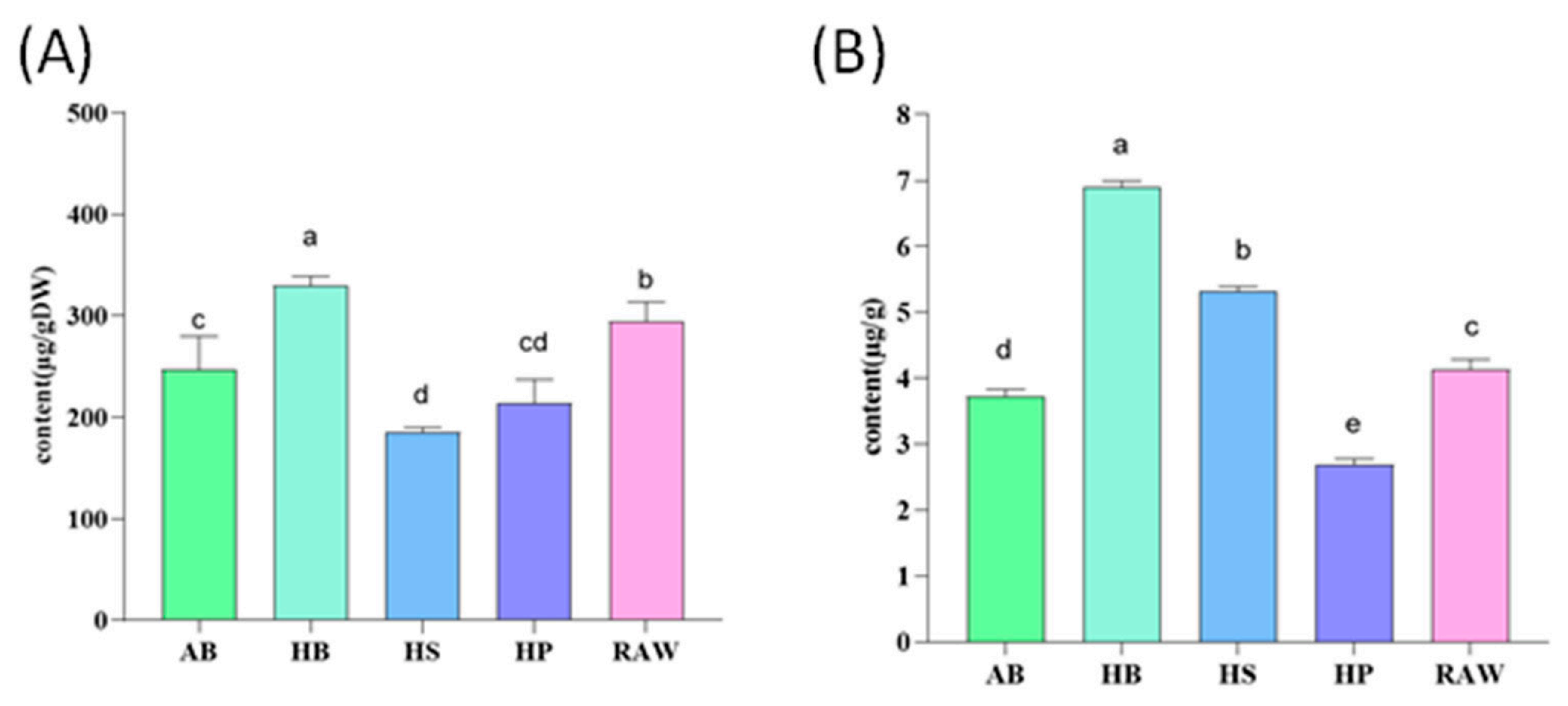
Influence of different hot processing on the total polyphenol and total carotenoid content of Fresh. Waxy Corn kernels. (**A**) Total polyphenol. (**B**) Total carotenoid. Different lowercase letters indicate significant differences between different groups (*p* < 0.05).

As illustrated in Figure 1A, the initial total phenolic content (TPC) of fresh waxy corn was 294.80 ± 18.74 μg/g. Compared to the untreated control, TPC significantly decreased by 16.27%, 36.98%, and 27.48% following AB, HS, and HP treatments, respectively. Conversely, HB treatment resulted in a 10.61% increase in TPC. Under these high-temperature and high-pressure conditions, the intermolecular interactions between polyphenols and cell wall polysaccharides were likely disrupted [19].

As depicted in Figure 1B, the initial total carotenoid content (TCC) of raw waxy corn was 4.14 ± 0.15 μg/g [20]. Following HB and HS treatments, TCC significantly increased by 39.92% and 22.22%, respectively, compared to the raw samples. In contrast, AB and HP treatments led to notable reductions of 10.14% and 35.02%, respectively. Specifically, HB treatment resulted in the highest TCC retention and liberation (*p* < 0.05), whereas HP treatment caused the most pronounced depletion (*p* < 0.05). These results indicate that the stability and extractability of carotenoids during thermal processing are dictated by both the specific processing parameters and the inherent characteristics of the corn matrix [21]. Thermal processing facilitates the structural disintegration of cell walls and the dissociation of carotenoid–protein complexes. This structural modification promotes the liberation of bound carotenoids from the food matrix, thereby enhancing their extractability and resulting in an increase in quantifiable content [22].

### 3.2. Analysis of Polyphenols and Carotenoids in Fresh Waxy Corn by Different Thermal Processing

**Figure 2:**
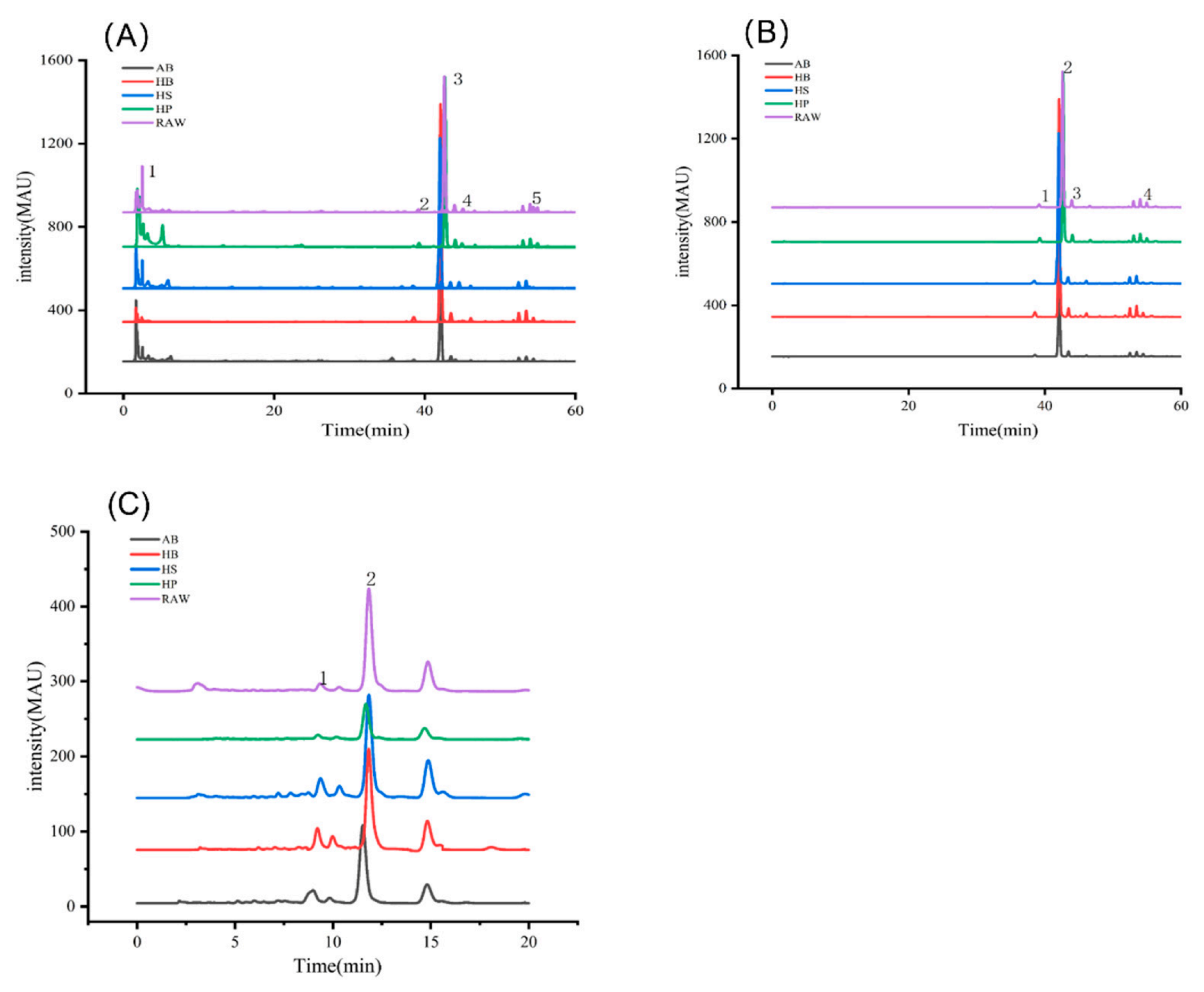
High-performance liquid chromatography of polyphenols and carotenoids in different hot-processed fresh waxy corn grains. (**A**) free phenol 1: Gall acid; 2: Procyanidin; 3: Rutin; 4: Ferulic acid; 5: Quercetin. (**B**) conjugated phenol 1: proanthocyanidins 2: Rutin; 3: Ferulic acid; 4: Quercetin. (**C**) Carotenoid 1: Lutein; 2: Zeaxanthin.

As shown in Figure 2A–C, five major free phenolic compounds and four major bound phenolic compounds were detected in fresh corn by HPLC analysis, including gallic acid, proanthocyanidins, rutin, ferulic acid, and quercetin as free phenolic compounds, and proanthocyanidins, rutin, ferulic acid, and quercetin as bound phenolic compounds. The two predominant carotenoids are lutein and zeaxanthin.

### 3.3. Analysis of Monomer Phenol of Fresh Waxy Corn Treated by Different Thermal Processing

While colorimetric assays provide a rapid estimation of total phenolics, they lack the specificity required to resolve changes in individual phenolic acid profiles under diverse thermal conditions. Consequently, HPLC was employed to achieve a more detailed characterization of the phenolic constituents. The chemical formulas, molecular weights, and concentrations of these compounds are summarized in Table 1, where total phenolics are defined as the cumulative sum of the free and bound fractions. Following the various thermal treatments, the levels of individual phenolic compounds exhibited distinct degrees of alteration. In corn treated with AB, the total phenolic acid content of the five monomers was lower than that of untreated corn. Rutin (4546.36 ± 93.77 μg/100 g) is the predominant phenolic compound in fresh waxy corn, followed by ferulic acid (1716.75 ± 72.36 μg/100 g), quercetin (1713.68 ± 177.43 μg/100 g), and procyanidins (73.79 ± 2.32 μg/100 g). As shown in Table 1, phenolic compounds mainly exist in free and bound forms. In thermally processed fresh waxy corn, more phenolic acids were detected in the bound form. After HB treatment, the contents of bound ferulic acid, quercetin, rutin, and procyanidins increased by 29.06%, 23.71%, 38.15%, and 20.82%, respectively, compared with untreated samples. Among the analyzed materials, bound phenolic compounds accounted for the highest proportion of total phenolic compounds, consistent with the findings of N’Dri et al. [23].

### 3.4. Analysis of Monomer Carotenoids in Fresh Waxy Corn by Different Thermal Processing

Table 2 presents the chemical formulas, molecular weights, and concentrations of the identified compounds, highlighting significant differences across thermal processing methods (*p* < 0.05). In untreated fresh waxy corn, lutein and zeaxanthin contents were 70.00 ± 0.02 and 157.88 ± 0.17 μg/100 g, respectively. Compared to the control, lutein levels decreased by 24.76% and 30.00% following AB and HP treatments, respectively, but increased by 11.02% under HS treatment. Similarly, zeaxanthin content decreased by 51.67% after AB treatment, whereas HS treatment resulted in a 10.48% increase.

HP treatment resulted in the lowest concentrations of lutein and zeaxanthin among all groups (*p* < 0.05), with zeaxanthin specifically exhibiting a marked reduction of 63.48%. Conversely, HB treatment significantly enhanced these levels (*p* < 0.05) by 42.31% and 40.43%, respectively, surpassing both the untreated and other thermal treatment groups.

### 3.5. Effects of Thermal Processing on the Antioxidant Activity of Fresh Waxy Corn

Table 3 illustrates that home cooking methods preserved the antioxidant activity of corn to varying degrees. Notably, the HB treatment exhibited the most potent antioxidant capacity, as indicated by significantly lower IC_50_ values across multiple assays. Specifically, in the DPPH assay, the carotenoids, free phenolics, and bound phenolics of HB-treated corn showed robust radical scavenging activities, with IC_50_ values of 1.16 ± 0.23, 0.40 ± 0.01, and 0.04 ± 0.01 mg/mL, respectively. Furthermore, the HB treatment yielded the highest ABTS^+^ scavenging efficiency for carotenoids (IC_50_ = 0.86 ± 0.07 mg/mL) and free phenolics (IC_50_ = 0.08 ± 0.01 mg/mL), while also demonstrating the strongest hydroxyl radical scavenging capacity for free phenolics (IC_50_ = 0.05 ± 0.01 mg/mL).

Thermal processing-induced alterations in bioactive compound levels can influence the resulting antioxidant capacity. Under high-temperature and high-pressure conditions, the rapid disruption of cereal cell walls may facilitate the release of antioxidants, potentially sustaining or enhancing the antioxidant potential. Specifically, AB treatment yielded the most potent scavenging activities for bound phenolics in corn, with IC_50_ values of 0.02 ± 0.01 mg/mL for ABTS^+^ and 0.08 ± 0.01 mg/mL for hydroxyl radicals. Meanwhile, HP treatment demonstrated the highest hydroxyl radical scavenging capacity for carotenoids (IC_50_ = 0.98 ± 0.07 mg/mL). Among the tested fractions, carotenoids displayed a relatively high ABTS^+^ scavenging efficiency, whereas free phenolics were more effective against hydroxyl radicals.

### 3.6. Correlation Between Antioxidant Activity and Active Substance of Fresh Waxy Corn

**Figure 3:**
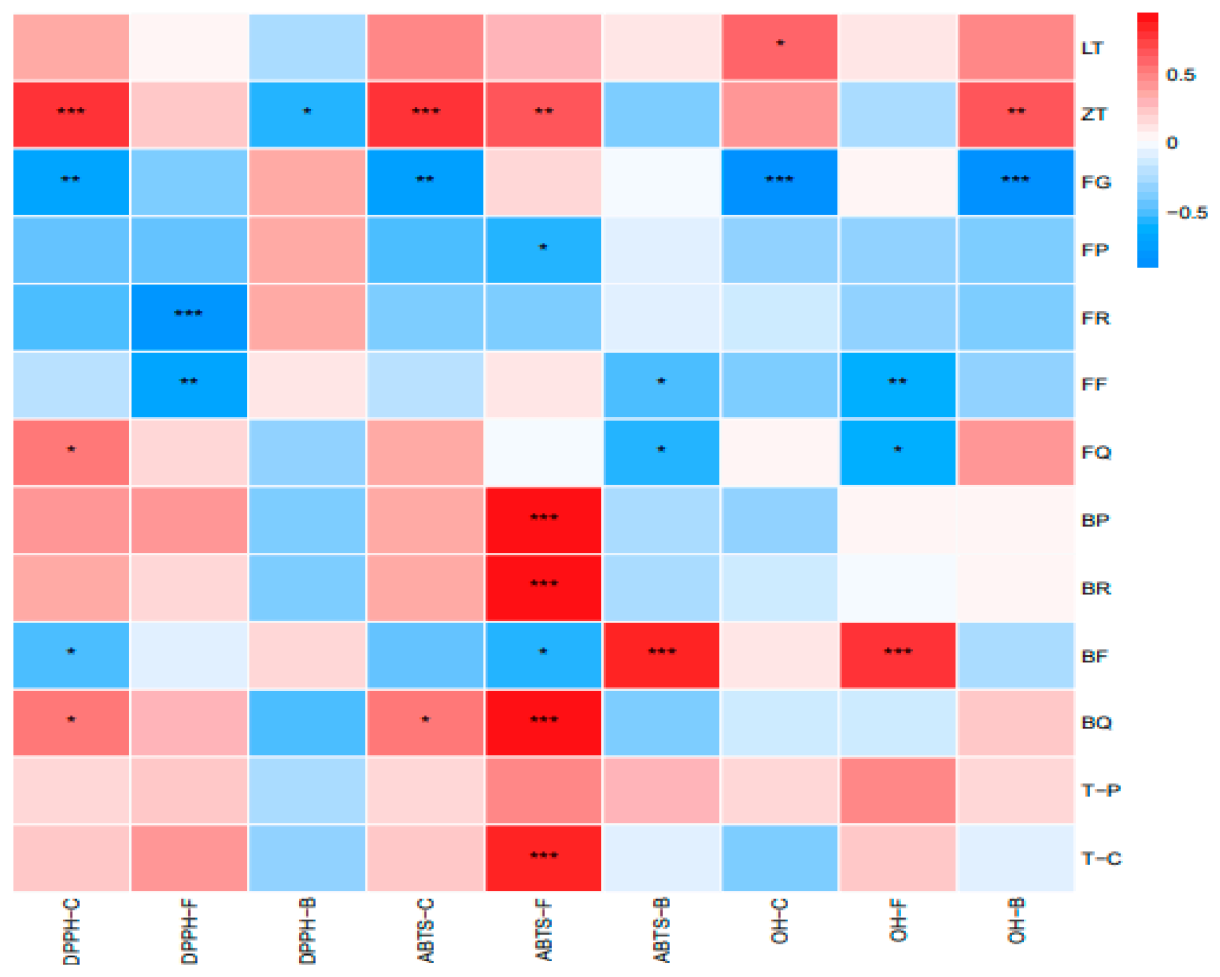
Correlation analysis between active substances and antioxidant activity of different thermally processed Fresh waxy corn. LT-lutein; ZT-zeaxanthin; FG-free gallic acid; FP-free proanthocyanidins; FR-free rutin; FF-free ferulic acid; FQ-free quercetin; BP-bound proanthocyanidins; BR- combined rutin; BF-bound ferulic acid; BQ-conjugated quercetin; TP-total phenol; TC-total carrot; DPPH.-C-carotenoid scavenging DPPH. Free radical ability; DPPH.-F free phenol scavenging DPPH. Free radical ability; DPPH.-B-binding phenol scavenging DPPH. Free radical ability; ABTS^+^ -C-carotenoid scavenging ability of ABTS^+^ free radicals; ABTS^+^-F free phenol scavenging ability of ABTS^+^ free radicals; ABTS^+^ -B-binding phenol scavenging ability of ABTS^+^ free radicals; -OH-C-carotenoid scavenging ability of hydroxyl free radicals; Hydroxyl radical scavenging ability of -OH-F free phenol; Hydroxyl radical scavenging ability of -OH-B-binding phenol. Gradient color barcodes at the right indicated the minimum value in blue and the maximum in red (for interpretation of the references to color in this Figure legend). Figure 3 indicates the r values of the correlation coefficient; * indicates *p* < 0.05; ** indicates *p* < 0.01; *** indicates *p* < 0.001.

Pearson correlation analysis was used to identify the major bioactive components contributing to the antioxidant activity of fresh waxy corn. As shown in Figure 3, the scavenging ability of carotenoids against DPPH radicals was significantly positively correlated with lutein content (*p* < 0.01). Similarly, the scavenging ability of carotenoids against ABTS^+^ radicals was also significantly positively correlated with lutein content (*p* < 0.01), while the scavenging ability of carotenoids against hydroxyl radicals was positively correlated with zeaxanthin content (*p* < 0.05). For free phenols, the scavenging ability against DPPH radicals was significantly positively correlated with the content of quercetin and ferulic acid (*p* < 0.01). The scavenging ability of free phenols against ABTS^+^ radicals was positively correlated with the content of free proanthocyanidins (*p* < 0.05). The total scavenging ability of free phenolics was significantly positively correlated with the content of free ferulic acid and quercetin (*p* < 0.01). Bound phenols showed a significant positive correlation between their scavenging ability against ABTS^+^ radicals and the content of bound ferulic acid (*p* < 0.01). In conclusion, free ferulic acid, quercetin, and proanthocyanidins were the main contributors to the antioxidant capacity of free phenolic compounds, whereas bound ferulic acid was the main contributor to the antioxidant capacity of bound phenolic compounds. Therefore, the contents of ferulic acid and zeaxanthin in fresh waxy corn exhibited the strongest correlation with total antioxidant capacity.

### 3.7. Principal Component Analysis

**Figure 4:**
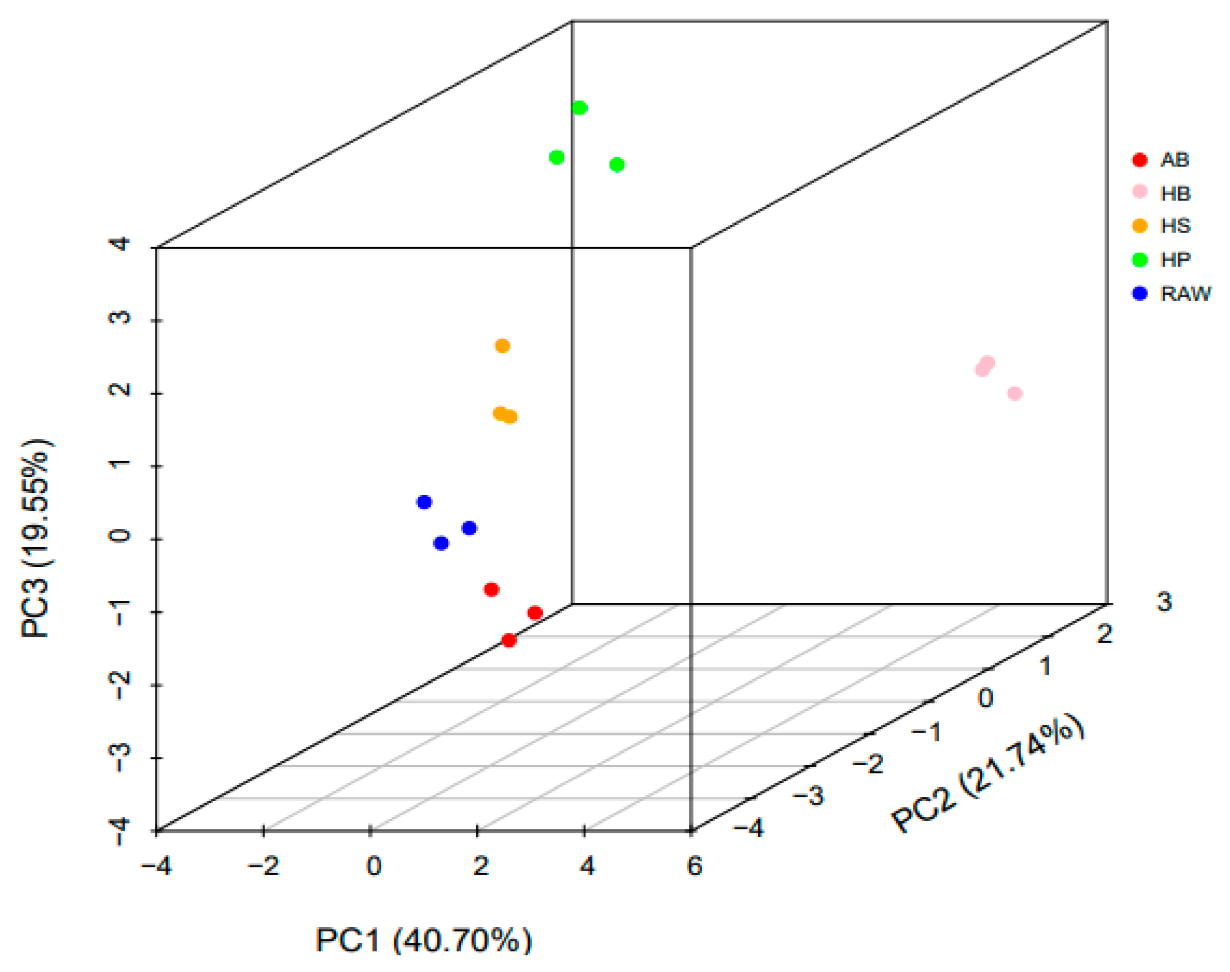
PCA score plot for properties of fresh waxy corn after processing by different thermal methods.

Principal component analysis (PCA) was performed to evaluate the effects of various thermal processing methods on the bioactive compounds and antioxidant activities of fresh waxy corn. As illustrated in Figure 4, the first three principal components (PC1, PC2, and PC3) accounted for 40.70%, 21.74%, and 19.55% of the total variance, respectively. The loading plots provided a robust basis for understanding the impact of thermal processing by reflecting the contribution and direction of each nutritional and functional index. Significant differences were observed among the four thermal treatment groups (*p* < 0.05). Specifically, the AB and HB treatments were positioned in the far-right and top regions of the plot, respectively, indicating distinct bioactive profiles between these two methods (*p* < 0.05). In contrast, the HS and AB treatments clustered closer to the non-thermal control and further from the HB group, suggesting that they had similar effects on the bioactive composition. The comprehensive scores for each treatment were calculated based on the principal component loading matrix, eigenvalues, variance, and cumulative contribution rates of each component (Supplementary Tables S3 and S4). As shown in Table 4, the HB treatment achieved the highest score, indicating its superior ability to facilitate the release and preservation of bioactive compounds. Consequently, the HB treatment was selected as the optimal method for subsequent in vitro digestion studies.

### 3.8. Effects of In Vitro Digestion on Polyphenol and Carotenoid Components of High Pressure Cooked Fresh Waxy Corn

**Figure 5:**
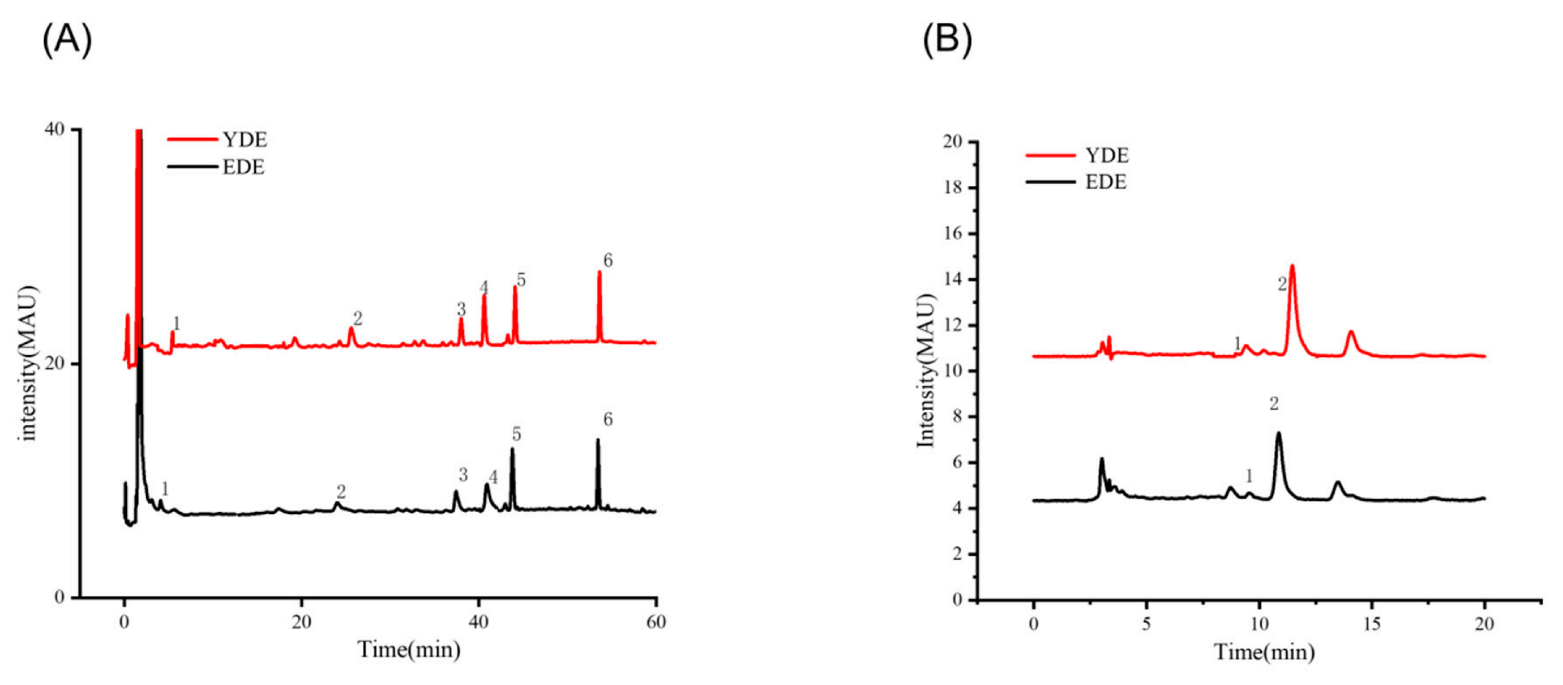
High-performance liquid chromatography of active substances in fresh waxy corn digested in vitro. (**A**) polyphenols. (**B**) carotenoids. (**A**) 1: gallic acid; 2: chlorogenic acid; 3: proanthocyanidins; 4: rutin; 5: Ferulic acid; 6: quercetin. (**B**) 1: Lutein; 2: zeaxanthin.

HPLC analysis of the micellar fraction from fresh waxy corn digesta identified two primary carotenoids (lutein and zeaxanthin) and six phenolic compounds (gallic acid, chlorogenic acid, proanthocyanidins, rutin, ferulic acid, and quercetin). As illustrated in Figure 5, in vitro gastrointestinal digestion not only altered the concentrations of these bioactive compounds but also led to the emergence of a novel absorption peak (peak 2, Figure 5A). Notably, several phenolic compounds detected post-digestion were absent in the raw material. This phenomenon can be attributed to the liberation of phenolics that were originally sequestered within the cell wall matrix, rendering them inaccessible to extraction prior to the digestive process.

### 3.9. Changes in the Content of Polyphenol and Carotenoid Monomers During In Vitro Digestion

**Figure 6:**
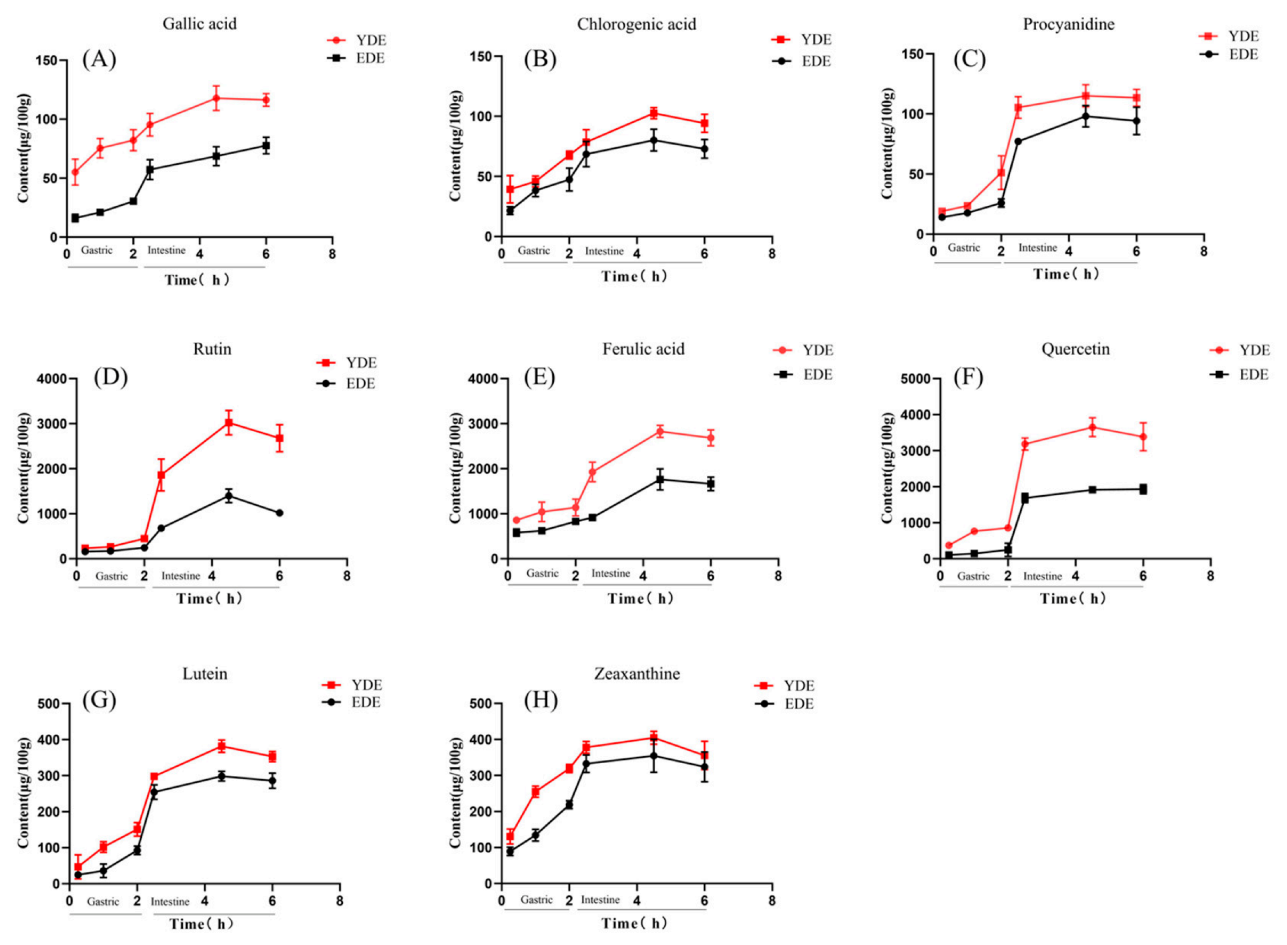
Changes in polyphenol monomer and carotenoid monomer content in digestive fluid micelles. (**A**) Gallic acid; (**B**) Chlorogenic acid; (**C**) Procyanidine; (**D**) Rutin; (**E**) Ferulic acid; (**F**) Quercetin; (**G**) Lutein; (**H**) Zeaxanthine.

The effect of in vitro digestion on polyphenols and carotenoids in high-pressure-cooked fresh waxy corn showed that the levels of various polyphenolic compounds gradually increased from the gastric phase to the intestinal phase and were higher than those of undigested polyphenols. The acidic environment (HCl, pH 1.0–3.0) and the action of digestive enzymes promoted the release of phenolic acids bound to solid food substrates. Ferulic acid is tightly bound by chemical bonds to proteins, starches, and dietary fibers. During digestion, the hydrolysis of proteins and starch releases the tightly bound ferulic acid, thereby increasing its content during the digestive process (Figure 6E) [24]. However, the levels of rutin and quercetin were the lowest in the in vitro digestive environment of the elderly (Figure 6D,F). The levels of phenolic compounds reached their maximum at 240 min of digestion and then decreased or stabilized, indicating that digestion was essentially complete at 240 min. The release of phenolic compounds in fresh waxy corn occurred mainly during the intestinal phase, which can be explained by the low solubility of polyphenols at low pH (1.0–3.0), with values increasing only when reaching the simulated small intestine. The additional digestion time (two hours) in the intestinal phase, the alkaline environment, the use of more digestive enzymes (trypsin), and the abundance of hydrolases in the small intestine all contribute to the release of phenolic compounds. Pancreatic enzymes and bile in the intestinal phase play an important role in the release of phenolic compounds, which can be released by binding to polysaccharides and fats. The degradation of plant tissue integrity during digestion facilitates the release and diffusion of phenolic compounds. In this study, the phenolic content within the elderly digestive model was significantly lower than that of the younger model. This disparity is likely attributable to the diminished gastrointestinal motility and reduced enzymatic activity characteristic of the elderly, which impair digestive capacity and the hydrolysis of the 32 identified phenolic compounds. Furthermore, the bioaccessibility of these compounds was evaluated by measuring their concentration in the micellar phase post-digestion, as this fraction represents the portion available for intestinal absorption.

The micellar phase obtained from the supernatant after centrifugation of the digestion products can be directly absorbed by the human body. The changes in the content of various carotenoid compounds under two different in vitro digestion conditions are shown in Figure 6G,H. During the simulated in vitro digestion, the content of lutein and zeaxanthin in the micelles gradually increased. The experiments showed that the bioactive compounds in heat-treated fresh waxy corn can be effectively absorbed and utilized by the human body during in vitro digestion. The levels of carotenoids gradually increased over time, reaching maximum release at 240 min. The oxygen atoms in the molecular structure of carotenoids such as lutein are mainly in the form of hydroxyl, methoxy, carbonyl, or epoxy groups, which makes lutein more polar and allows it to dissolve more effectively in the bile salt-containing intestinal phase, resulting in a significant increase in lutein content during the in vitro digestion phase (*p* < 0.05) [25]. During simulated in vitro digestion, zeaxanthin enveloped by zeaxanthin–starch and zeaxanthin–protein complexes in fresh corn flour was dissociated under the action of α-amylase, gastric juice, bile, and trypsin, resulting in a significant increase in zeaxanthin content (*p* < 0.05).

### 3.10. Effect of High-Pressure Boiling on the Bioaccessibility of Polyphenols and Carotenoids in Fresh Waxy Corn

**Figure 7:**
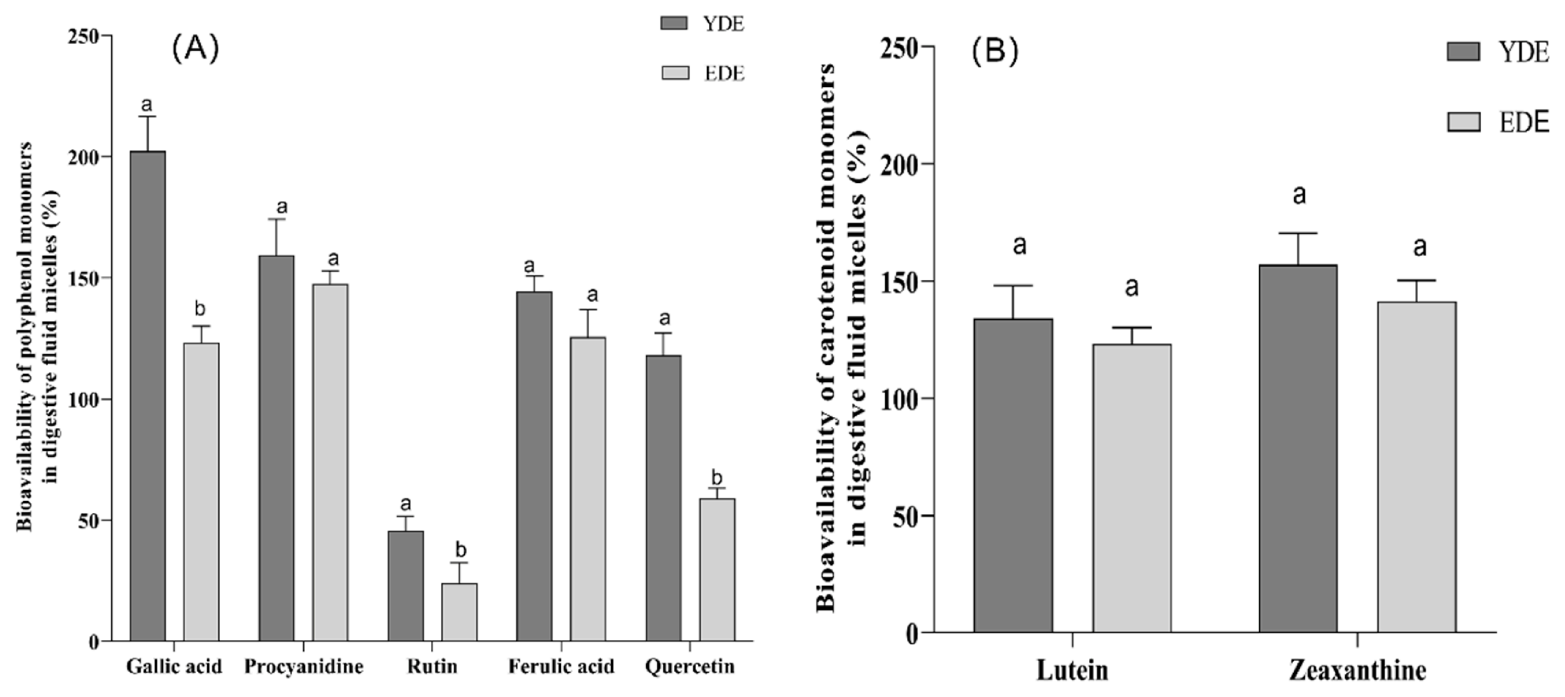
Bioaccessibility of polyphenol and carotenoid monomers for gastrointestinal digestion in vitro. (**A**) polyphenols. (**B**) carotenoid. Different lowercase letters in data labeling indicate significant differences among different groups (*p* < 0.05). Young adults in vitro digestive environment (YDE), in vitro digestive environment for the elderly (EDE).

The bioaccessibility of bioactive compounds in fresh waxy corn was evaluated using an in vitro simulated gastrointestinal digestion model (Figure 7A,B). Following digestion, the average bioaccessibility of the quantified phenolic compounds, ranked from highest to lowest, was as follows: YDE group: gallic acid (202.28 ± 14.29%) > proanthocyanidins (159.37 ± 14.85%) > ferulic acid (144.50 ± 6.33%) > quercetin (118.05 ± 9.27%) > rutin (45.61 ± 6.00%); EDE group: proanthocyanidins (147.61 ± 5.29%) > ferulic acid (125.59 ± 11.30%) > gallic acid (123.15 ± 6.90%) > quercetin (59.11 ± 4.10%) > rutin (23.95 ± 8.40%). Rutin exhibited the lowest bioaccessibility in both groups (45.61 ± 6.00% in YDE vs. 23.95 ± 8.40% in EDE). Notably, the bioaccessibility of gallic acid, rutin, and quercetin was significantly reduced in the simulated elderly digestive environment (EDE) compared to that in the young digestive environment (YDE). The most pronounced decrease was observed for rutin, which declined by approximately 47% (from 45.61 ± 6.00% to 23.95 ± 8.40%) (Figure 7A).

Upon consumption, fresh waxy corn kernels are masticated and mixed with salivary amylase to form a bolus, which then undergoes sequential digestion in the gastric and intestinal phases. Throughout this gastrointestinal transit, complex chemical reactions and enzymatic hydrolysis modulate the bioaccessibility and bioactivity of the phenolic compounds. While a portion of these cereal polyphenols is absorbed within the small intestine, the remaining compounds interact directly with the intestinal epithelium. These interactions enhance intestinal health by reinforcing barrier function through antioxidant and anti-inflammatory pathways, as well as by promoting mucus production.

As shown in Figure 7B, the average bioavailability of different carotenoid compounds after 34 in vitro-simulated gastrointestinal digestions, from highest to lowest, was as follows: zeaxanthin (YDE: 157.06 ± 13.35%; EDE: 141.46 ± 8.86%) > pyrite (YDE: 134.13 ± 13.88%; EDE: 123.24 ± 6.92%). High-temperature treatment can disrupt plant cell walls and plastid membranes, favoring the translocation of carotenoids into the micellar phase. The action of bile salts and pancreatic lipase can also significantly increase the digestion and absorption rate of carotenoids. Therefore, during in vitro gastrointestinal digestion, carotenoids are released from the micelles into the digestive fluid and can be absorbed by intestinal cells.

## 4. Discussion

The HPLC analysis revealed that rutin, gallic acid, and ferulic acid are the primary phenolics in fresh waxy corn, consistent with the high antioxidant distribution noted by Cai [19]. Thermal processing, particularly high-pressure treatments (HB and HS), enhanced free phenolic levels by disrupting the lignocellulosic matrix and releasing “trapped” compounds [26]. This reduction is likely attributable to the generation of steam-induced free radicals, which facilitate the formation of reactive oxygen species (ROS) such as H_2_O_2_, thereby triggering the oxidative degradation of phenolic compounds [17]. While atmospheric boiling (AB) led to some leaching, HS better preserved bound ferulic acid, potentially due to reduced oxygen exposure and a hydrothermal “seal” effect [27]. Furthermore, HB treatment may have disrupted cellular structures, facilitating the release of bound phenolics and thereby leading to an overall increase in measurable total phenolic content (TPC) [12]. In addition to the aforementioned free and bound phenolic compounds, fresh waxy corn also contains p-coumaric acid, coumarin, coumaric acid, and dihydroluteolin [19]. The thermal processing of fresh waxy corn significantly altered its phytochemical profile, with high-pressure boiling (HB) emerging as the most effective method for enhancing both nutritional and functional properties. While atmospheric boiling (AB), steaming (HS), and high-speed pulping (HP) generally reduced TPC due to thermal degradation and the leaching of water-soluble phenolics [28], or oxidative polymerization triggered by mechanical shearing in the HP group, HB treatment induced a 10.61% increase in TPC. This enhancement is primarily attributed to the “matrix release” effect, where the synergistic application of high temperature and pressure facilitates the cleavage of ester and ether bonds that anchor bound phenolics (e.g., ferulic acid) to cell wall polysaccharides [29]. These variations may be attributed to heat transfer during thermal processing, which progresses from the surface to the inner tissue layers, inducing tissue softening and cell separation. Thermal processing facilitates the disintegration of plant cell walls, enhancing the liberation of both free and bound phenolic compounds from the corn matrix. While bound phenolics are typically conjugated to macromolecules, such as polysaccharides, proteins, starch, and dietary fiber, thermal treatments can disrupt these linkages. However, the high-moisture environment characteristic of certain cooking methods may simultaneously accelerate the oxidative degradation of free phenolics, leading to a significant reduction in their measurable concentration. The heat generated during thermal processing can induce structural changes in phenolic compounds, which in turn may affect their bioactivity and antioxidant activity to some extent [17]. This process compromises plasma membrane integrity and disrupts cellular compartments. Notably, compared to atmospheric boiling, the use of saturated steam under pressure in an autoclave appears more effective in facilitating the liberation of bioactive compounds from the waxy corn matrix. High-pressure treatment not only disrupts cell walls but also dissolves cellular materials into the serum phase, facilitating the extraction of carotenoids [4]. Similarly, TCC increased significantly under HB and HS treatments (39.92% and 22.22%, respectively), as thermal energy denatures sequestered protein–lipid complexes and softens cell wall pectin, thereby improving the extractability of lipophilic carotenoids [30]. Conversely, the reduction in TCC in the AB and HP groups underscores the vulnerability of the carotenoid polyene chain to oxidative degradation and trans-cis isomerization when exposed to open-system heating or air incorporation [31]. The divergent responses of hydrophilic phenolics and lipophilic carotenoids, particularly the TPC decrease versus TCC increase under HS, highlight their distinct chemical stabilities; however, the simultaneous enhancement of both components under HB provides a robust theoretical basis for utilizing high-pressure technology to produce nutrient-dense, functional waxy corn products, challenging the traditional view that thermal processing inevitably diminishes the nutritional value of vegetables.

The lower release of phenolic acids (e.g., ferulic acid and gallic acid) in EDE can be primarily attributed to the reduced secretion of pepsin and pancreatic enzymes. According to the modified INFOGEST protocol for elderly populations [18] gastric pepsin activity and intestinal pancreatin levels are significantly lower in the elderly to simulate reduced secretory capacity. Since ferulic acid in waxy corn is predominantly ester-linked to cell wall arabinoxylans and cross-linked with proteins [32], the weakened proteolysis and amylolysis in EDE likely result in incomplete disintegration of the corn starch–protein matrix. This prevents the “liberation” of bound phenolics into the aqueous phase. This finding is consistent with the study by Hernández-Olivas, Ever et al. [33], which reported that decreased masticatory performance and enzymatic activity in the elderly significantly delay the bolus breakdown, thereby reducing the surface area available for further chemical extraction. The most striking difference was observed for rutin, which showed a 47% reduction in bioavailability in the EDE group. This can be explained through two lenses: solubility and chemical stability. Elderly digestive models often feature a higher gastric pH (pH 3.0–4.0 instead of pH 2.0) and a slower transition to the alkaline intestinal environment. While rutin is relatively stable under acidic conditions, its transition into the micellar phase is highly dependent on the surfactant effect of bile salts. Kosmerl et al. [34] demonstrated that lower bile salt concentrations (a hallmark of the elderly digestive system) lead to poor micellarization of flavonols. Furthermore, the prolonged residence at time in the slightly higher gastric pH of the elderly may facilitate the auto-oxidation or degradation of sensitive polyphenols like quercetin before they reach the absorptive site of the small intestine, a phenomenon also noted by Sams [35] in their study on the bioaccessibility of antioxidants in Mediterranean diets.

## 5. Conclusions

This study investigated the effects of different thermal processing methods on the bioactive compounds in fresh waxy corn, identifying lutein, zeaxanthin and other carotenoids as well as gallic acid, proanthocyanidins, and other phenolic compounds as the major active components in the kernels. The results showed that HB treatment significantly increased the content of conjugated phenolics and carotenoids, with ferulic acid, quercetin, rutin, proanthocyanidins, lutein, and zeaxanthin increasing by 29.06%, 23.71%, 38.15%, 20.82%, 42.31% and 40.43%, respectively. The strong correlation between ferulic acid and zeaxanthin content and total antioxidant capacity indicated that HB treatment was the optimal processing method. In vitro digestion experiments showed that the content of carotenoids and phenolic compounds in the micelles of high-pressure-cooked fresh waxy corn increased during digestion. However, the bioavailability of gallic acid, rutin, and quercetin was significantly reduced in the gastrointestinal digestive environment of the elderly, especially for rutin. Strategies to enhance its bioaccessibility warrant further investigation; for instance, encapsulation techniques could be employed to facilitate targeted delivery to the small intestine, thereby maximizing its absorption. Furthermore, compared to the simulated digestion model of young adults, the bioaccessibility and potential absorption of rutin, gallic acid, and quercetin were notably lower in the elderly model. Consequently, future research should explore the development of targeted nutritional fortifiers specifically designed for the elderly population.

## Figures and Tables

**Table 1:** Changes in polyphenol monomer content in fresh waxy by different thermal processing.

Ingredient	Code	Formula	Molecular	λ (nm)	Free Phenol (μg/100 g)	Bound Phenol (μg/100 g)	Total Phenol (μg/100 g)
Gallic acid	AB	C_7_H_6_O_5_	170.12	280	101.42 ± 16.87 ^c^	-	101.42 ± 16.87 ^c^
	HB				64.07 ± 2.45 ^c^	-	64.07 ± 2.45 ^c^
	HS				175.90 ± 6.05 ^b^	-	175.90 ± 6.05 ^b^
	HP				262.30 ± 9.04 ^a^	-	262.30 ± 9.04 ^a^
	RAW				124.52 ± 6.18 ^c^	-	124.52 ± 2.31 ^c^
Procyanidine	AB	C_30_H_12_O_6_	594.52		40.69 ± 0.81 ^b^	22.47 ± 0.09 ^e^	63.16 ± 0.90 ^d^
	HB				16.65 ± 0.19 ^d^	39.10 ± 0.61 ^a^	55.75 ± 0.70 ^e^
	HS				37.72 ± 0.69 ^c^	28.14 ± 0.62 ^d^	65.86 ± 2.59 ^c^
	HP				43.54 ± 0.16 ^a^	32.74 ± 0.25 ^b^	76.28 ± 1.98 ^a^
	RAW				42.83 ± 0.33 ^a^	30.96 ± 0.09 ^c^	73.79 ± 2.32 ^b^
Rutin	AB	C_7_H_6_O_5_	610.50		117.95 ± 5.86 ^b^	3225.03 ± 22.75 ^e^	3342.98 ± 64.03 ^e^
	HB				19.28 ± 1.04 ^e^	7242.85 ± 24.11 ^a^	7262.13 ± 184.43 ^a^
	HS				37.72 ± 0.69 ^d^	5512.21 ± 4.52 ^b^	5549.93 ± 167.96 ^b^
	HP				121.52 ± 12.01 ^a^	5183.83 ± 34.60 ^c^	5305.35 ± 168.28 ^c^
	RAW				66.34 ± 0.29 ^c^	4480.02 ± 166.52 ^d^	4546.36 ± 93.77 ^d^
Ferulic acid	AB	C_7_H_6_O_5_	170.12	280	54.85 ± 0.44 ^d^	878.04 ± 21.48 ^e^	932.85 ± 19.59 ^e^
	HB				51.11 ± 0.64 ^e^	2162.87 ± 57.92 ^a^	2213.98 ± 159.42 ^a^
	HS				81.03 ± 0.41 ^a^	1580.66 ± 47.46 ^c^	1661.69 ± 39.95 ^c^
	HP				71.36 ± 0.52 ^b^	1130.89 ± 21.41 ^d^	1202.25 ± 76.98 ^d^
	RAW				66.6 ± 1.06 ^c^	1650.15 ± 103.29 ^b^	1716.75 ± 72.36 ^b^
Quercetin	AB	C_30_H_12_O_6_	594.52	280	151.07 ± 33.49 ^b^	835.66 ± 30.18 ^d^	986.73 ± 20.21 ^e^
	HB				109.90 ± 4.70 ^d^	2355.49 ± 55.32 ^a^	2355.49 ± 26.06 ^a^
	HS				117.27 ± 5.06 ^c^	1664.19 ± 39.81 ^b^	1781.46 ± 75.07 ^c^
	HP				216.08 ± 3.03 ^a^	1589.63 ± 44.78 ^c^	1805.71 ± 73.06 ^b^
	RAW				105.74 ± 7.42 ^d^	1670.94 ± 120.29 ^b^	1713.68 ± 177.43 ^d^

Different lowercase letters indicate that there is a significant difference between different groups (*p* < 0.05), and - indicates that there is no detection.

**Table 2:** Changes in carotenoid monomer content in fresh waxy by different thermal processing.

Ingredient	Code	Formula	λ (nm)	Molecular	Content (μg/100 g)
Lutein	AB	C_72_H_116_O_4_	450	568.85	52.67 ± 8.96 ^c^
	HB				121.33 ± 11.42 ^a^
	HS				78.67 ± 4.04 ^b^
	HP				49.00 ± 3.13 ^e^
	RAW				70.00 ± 6.52 ^d^
Zeaxanthine	AB				73.41 ± 4.17 ^d^
	HB				265.05 ± 14.05 ^a^
	HS				176.36 ± 10.11 ^b^
	HP				57.65 ± 5.93 ^e^
	RAW				157.88 ± 14.15 ^c^

Different lowercase letters in data labeling indicate significant differences among different groups (*p* < 0.05).

**Table 3:** Analysis of antioxidant capacity.

IC_50_ (mg/mL)	DPPH			ABTS^+^			-OH		
	Carotenoid	Free Phenol	Bound Phenol	Carotenoid	Free Phenol	Bound Phenol	Carotenoid	Free Phenol	Bound Phenol
AB	1.81 ± 0.11 ^b^	0.71 ± 0.02 ^c^	0.07 ± 0.01 ^b^	1.16 ± 0.12 ^b^	0.19 ± 0.07 ^a^	0.02 ± 0.01 ^d^	1.89 ± 0.13 ^a^	0.06 ± 0.01 ^cd^	0.08 ± 0.01 ^d^
HB	1.16 ± 0.23 ^d^	0.40 ± 0.01 ^e^	0.04 ± 0.01 ^cd^	0.86 ± 0.07 ^e^	0.08 ± 0.01 ^e^	0.05 ± 0.01 ^c^	1.78 ± 0.08 ^b^	0.05 ± 0.01 ^d^	0.10 ± 0.01 ^cd^
HS	1.78 ± 0.09 ^c^	0.56 ± 0.03 ^d^	0.05 ± 0.02 ^c^	1.09 ± 0.03 ^d^	0.17 ± 0.04 ^b^	0.07 ± 0.01 ^b^	1.14 ± 0.12 ^d^	0.16 ± 0.08 ^a^	0.12 ± 0.02 ^b^
HP	1.94 ± 0.16 ^a^	0.89 ± 0.07 ^a^	0.13 ± 0.06 ^a^	1.21 ± 0.02 ^c^	0.1 ± 0.01 ^d^	0.03 ± 0.01 ^d^	0.98 ± 0.07 ^e^	0.11 ± 0.01 ^b^	0.09 ± 0.01 ^cd^
RAW	1.20 ± 0.04 ^e^	0.76 ± 0.02 ^b^	0.03 ± 0.01 ^d^	1.28 ± 0.01 ^a^	0.14 ± 0.01 ^c^	0.09 ± 0.01 ^a^	1.49 ± 0.13 ^c^	0.07 ± 0.03 ^c^	0.21 ± 0.01 ^a^

Different lowercase letters in data labeling indicate significant differences among different groups (*p* < 0.05).

**Table 4:** Comprehensive scores of the feature vectors of the four processing methods.

Process	F1	F2	F3	F4	Synthesis Score	Ranking
AB	−0.987792234	0.475410404	−1.574719167	−0.065173227	−0.613495315	3
HB	1.698544371	0.884643773	−0.185721011	−0.01806441	0.845391379	1
HS	−0.155503934	−0.607471512	0.403117078	1.74607684	0.069935987	2
HP	−1.028277575	−1.586597987	−0.014934927	−1.028277575	−0.876175198	4

## Data Availability

The original contributions presented in the study are included in the article and Supplemental Materials. Further inquiries can be directed to the corresponding authors.

## Supplementary Materials

The following supporting information can be downloaded at: https://www.mdpi.com/article/10.3390/foods15152648/s1, Figure S1: Changes of carotenoid monomer content in digestive fluid micelles; Table S1: Simulated In vitro digestion electrolyte solution ratio; Table S2: Preparation of stock solutions of simulated digestion fluids; Table S3: Principal component load matrix; Table S4: Eigenvalue variance and cumulative contribution rate of each component.
