# Supplementary material for: Effects of Thermal Processing on the Contents and Bioaccessibility of Polyphenols and Carotenoids in Fresh Waxy Corn
Source: Foods. 2026 Jul 28;15(15):2648. doi: 10.3390/foods15152648 (PMC13466067; doi:10.3390/foods15152648)
Supplement: Supplementary file 1 [file foods-15-02648-s001.zip › foods-4399846-supplementary.pdf]

Supplementary Materials

Table S1 Simulated *In vitro* digestion electrolyte solution ratio

Parameters of *In vitro* tsampa digestion	Young adults	Elderly
Sample	5 g	5 g
$Cacl_2$（oral）	1.5 mM	1.5 mM
$Cacl_2$（gastric）	0.15 mM	0.15 mM
$Cacl_2$（intestine）	0.6 mM	1.2 mM
Salivary α-amylase	150 U/mL SSF	225 U/mL SSF
Pepsin	4000 U/mL SGF	3000 U/mL SGF
Pig bile salt	10 mM/L SIF	10 mM/L SIF
Pancreatin	200 U/mL SIF	100 U/mL SIF
Trypsin	100 U/mL SIF	100 U/mL SIF
Fasting pH	1.6	3
Crawling speed	1 mol/L, 0–10 min: 0.5 mL/min	0.5 mol/L, 0–10 min: 0.5 mL/min

Table S2 Preparation of stock solutions of simulated digestion fluids.

The volumes are calculated for a final volume of 500 mL for each simulatedfluid. We recommend to make up the stock solution with distilled water to 400 mL instead, i.e. 1.25* concentrate, for storage at -20℃. In theExperimental section, these 1.25* concentrates are referred to as Simulated Salivary Fluid (SSF), Simulated Gastric Fluid (SGF) and Simulated.Intestinal Fluid (SIF) electrolyte stock solutions. The addition of enzymes, bile salts, Ca2+ solution etc. and water will result in the correct electrolyte concentration in the final digestion mixture. $CaCl_2(H_2O)_2$ is not added to the electrolyte stock solutions as precipitation may occur.Instead, it is added to the final mixture of simulated digestion fluid and fooda.

Constituent	Stock conc.		SSF		SGF		SIF	
			Vol. of stock	Conc. in SSF	Vol. of stock	Conc. in SGF	Vol. of stock	Conc. in SIF
	g/L	mol/L	mL	mmol/L	mL	mmol/L	mL	mmol/L
KCl	37.3	0.5	15.1	15.09	6.9	6.9	6.8	6.8
KH_2PO_4	68	0.5	3.7	1.35	0.9	0.9	0.8	0.8

Constituent	Stock conc.		SSF		SGF		SIF	
			Vol. of stock	Conc. in SSF	Vol. of stock	Conc. in SGF	Vol. of stock	Conc. in SIF
			Vol. of stock	Conc. in SSF	Vol. of stock	Conc. in SGF	Vol. of stock	Conc. in SIF
$NaHCO_3$	84	1	6.8	13.68	12.5	25	42.5	85
NaCl	117	2	-	-	11.8	47.2	9.6	38.4
$MgCl_2(H_2O)_6$	30.5	0.15	0.5	0.15	0.4	0.12	1.1	0.33
$(NH_4)_2CO_3$	48	0.5	0.06	0.06	0.5	0.5	-	-
NACL	117	2	-	-	11.8	47.2	9.6	38.4

Table S3 Principal component load matrix

	Principal component			
Compounds	1	2	3	4
LT	0.20932	0.11826	-0.17494	0.40938

ZT	0.3171	-0.04945	-0.02399	0.18655
FG	-0.16971	0.11963	0.38968	0.01168
FP	-0.29017	-0.1943	0.07076	-0.11828
FR	-0.22258	-0.08269	0.09322	0.44067
FF	-0.13172	-0.20823	0.30716	0.28947
FQ	0.02423	-0.36291	0.0199	-0.37662
BP	0.2675	0.11115	0.2417	-0.13839
BR	0.27296	0.12985	0.21525	0.14262
BF	-0.12406	0.30138	-0.24797	0.05688
BQ	0.29434	0.02969	0.22134	0.02391
T-P	0.22509	0.28488	-0.03838	0.16518
T-C	0.24964	0.19466	0.22276	-0.13326
DPPH.-C	0.2591	-0.24892	-0.10105	-0.0525
DPPH.-F	0.1552	0.08958	-0.1166	-0.4517
DPPH.-B	-0.20492	0.11814	0.03612	0.07311
ABTS+-C	0.2525	-0.22819	-0.12279	0.05956
ABTS+-F	0.24441	0.03331	0.31002	0.02818
ABTS+-B	-0.09661	0.34535	-0.21767	-0.07462
-OH-C	0.09791	-0.1234	-0.4083	0.1865
-OH-F	-0.03539	0.42347	-0.12503	-0.12745
-OH-B	0.19289	-0.24223	-0.27601	0.04736

Table S4 Eigenvalue variance and cumulative contribution rate of each component

PC	Eigenvalue	Variance percentage	Accumulate
1	8.95446	40.70%	40.70%
2	4.78359	21.74%	62.45%
3	4.30177	19.55%	82.00%
4	2.34936	10.68%	92.68%

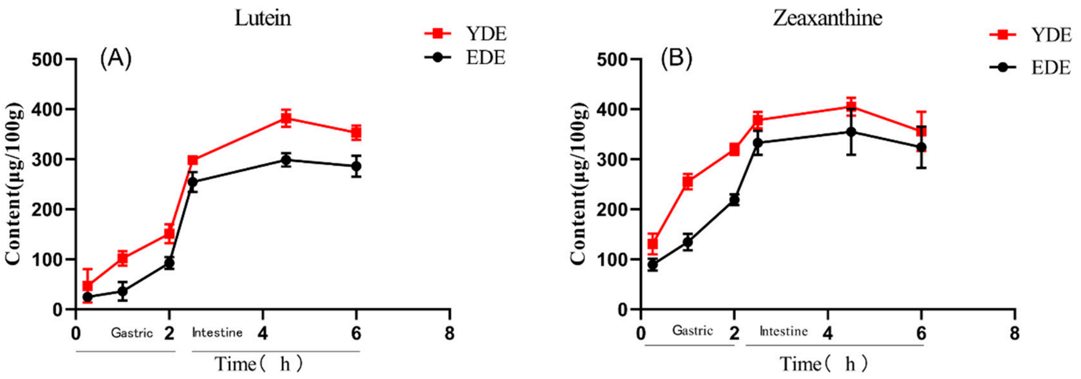

Figure S1 Changes of carotenoid monomer content in digestive fluid micelles
